# Deciphering the Genetic Basis of Allelopathy in *japonica* Rice Cultivated in Temperate Regions Using a Genome-Wide Association Study

**DOI:** 10.1186/s12284-024-00701-3

**Published:** 2024-03-26

**Authors:** Julia García-Romeral, Raúl Castanera, Josep Casacuberta, Concha Domingo

**Affiliations:** 1 Departamento del Arroz, Centro de Genómica, Instituto Valenciano de Investigaciones Agrarias (IVIA), Carretera CV-315. km 10.7, 46113 Moncada, Valencia Spain; 2 Centre for Research in Agricultural Genomics (CRAG) CSIC-IRTA-UAB-UB, Campus Universitat Autònoma de Barcelona (UAB), Bellaterra (Cerdanyola del Vallés), C/de la Vall Moronta, CRAG Building, 08193 Barcelona, Spain

**Keywords:** Allelopathy, QTL, Genome Wide Association Study (GWAS)

## Abstract

Allelopathy has been considered as a natural method of weed control. Despite the nature of allelochemical compounds has been studied, little is known about the genetic basis underlying allelopathy. However, it is known that rice exhibits diverse allelopathic potentials across varieties, and breeding for rice plants exhibiting allelopathic potential conferring an advantage against weeds in paddy fields would be highly desirable. Knowledge of the gene factors and the identification of the genomic regions responsible for allelopathy would facilitate breeding programs. Taking advantage of the existing genetic diversity in rice, particularly in temperate *japonica* rice, we conducted a comprehensive investigation into the genetic determinants that contribute to rice allelopathy. Employing Genome-Wide Association Study, we identified four Quantitative Trait Loci, with the most promising loci situated on chromosome 2 and 5. Subsequent inspection of the genes located within these QTLs revealed genes associated with the biosynthesis of secondary metabolites such as *Phenylalanine Ammonia Lyase* (*PAL*), a key enzyme in the synthesis of phenolic compounds, and two genes coding for R2R3-type MYB transcription factors. The identification of these two QTLs associated to allelopathy in rice provides a useful tool for further exploration and targeted breeding strategies.

**Supplementary Information:**

The online version contains supplementary material available at 10.1186/s12284-024-00701-3.

## Background

Rice is a global staple food and high yields are needed to sustain a large population. It is also important to facilitate a sustainable crop and to increase the incomes of farmers and the economic profitability of the crop. Weeds, in their competition for nutrients, moisture, and light, diminish rice yields. The management of weed control involves the application of herbicides, which is environmentally unsustainable, and requires a high amount of manual labor, which is costly and profitless. The quest for an economically and environmentally sustainable approach to weed control remains a challenge. In this context, allelopathy can be considered as an environmentally friendly practice for weed control. Allelopathy has been described as the ability of an organism to affect growth, survival, and reproduction of other organisms through the secretion of allelochemicals or their release into the environment (Rice 1984). Allelopathy has been observed in numerous plant species, and its potential impact is well-documented. In fact, the efficiency of using straw and hulls from certain crops, including rice, as mulch for weed control has been successfully demonstrated (Khamare et al. 2022). Among others, barnyardgrass (*Echinochloa crus-galli*) is a globally extended harmful weed, frequently found in paddy fields, that produces field losses (Oerke and Dehne 2004) and, worriedly, the appearance of resistance to herbicides has been frequently observed (Amaro-Blanco et al. 2021).

In rice, allelopathic effects have been described in certain varieties (Dilday et al. 1994). Some varieties can synthesize and release chemical compounds into the environment that can affect the growth and development of neighbouring plants by interfering with their physiological processes, such as seed germination, root elongation, and nutrient uptake. Their inhibitory effect on weeds, such as ducksalad (*Heteranthera limosa*) or barnyardgrass, has been reported (Dilday et al. 1994). In the late 1980s, hundreds of accessions from germplasm collections were examined in field experiments for their allelopathic potential towards ducksalad, redstem (*Ammannia coccinea Rottb*.), lettuce (*Lactuca sativa* L.) and barnyard grass among others (Dilday et al. 1994; Hassan et al. 1998). More recently, bioassays were developed to reduce environmental factors, and hundreds of accessions were also screened in the laboratory (Kohli et al. 2008). As a result, several accessions with strong allelopathic potential have been identified, as is the case of PI213777 (Dilday et al. 1994).

Several allelochemicals have been isolated from plants and root exudates of different plant species and many studies have been performed to find the nature and the effect of such compounds in the rice with barnyardgrass interaction (Macías et al. 2007). Most reported allelochemicals in rice are secondary plant metabolites, including fatty acids, indoles, momilactones, phenolics and terpenes (Seal et al. 2004; Khanh et al. 2007; Kato-Noguchi and Peters 2013). Transcriptomic studies have also been conducted in the interaction of rice with barnyardgrass and differential expressed genes associated with momilactone and phenolic acid biosynthesis were found to be up-regulated in a quick allelopathic response (Song et al. 2008; Sultana et al. 2023). But no candidate genes have already been reported.

Secondary metabolites are mainly derived from the phenylpropanoid metabolic and isoprenoid pathways and their associated branches (Fang et al. 2020). Phenylalanine ammonia lyase (PAL) is an initial key enzyme in the biosynthesis of phenolic compounds, catalysing the conversion of phenylalanine to cinnamic acid, which is a precursor for the synthesis of allelopathic phenolic compounds such as ferulic acid and p-coumaric acid. The role of PAL in the regulation of chemical induction in allelopathy has been reported previously and the up-regulated expression of *PAL* is associated with the enhanced inhibitory effect of allelopathy grown with barnyard (Zhang et al. 2019b).

Momilactones A and B were first isolated from rice husk, as growth inhibitors (Takahashi et al. 1976) and more recently they have been found in root exudates of allelopathic cultivars as PI312777 and Koshihikari (Kong et al. 2004; Kato-Noguchi et al. 2008). Momilactones have been considered phytoalexins in rice protection against fungi (Cartwright et al. 1977; Cartwright et al. 1981). Later on, their ability to inhibit weed growth was demonstrated (Kato-Noguchi et al. 2010). The biosynthetic pathway of momilactones is well known (Kato-Noguchi and Peters 2013), and knock-outs of relevant genes participating in the diterpertene synthesis pathway such as copalyl diphosphate synthase 4 (OsCPS4) or kaurene synthase-like 4 (OsKSL4) result in reduced allelopathic activity of rice in barnyard (Xu et al. 2012). Two gene clusters have been identified in the synthesis of momilactones (Xu et al. 2012; Kato-Noguchi and Peters 2013).

The role of phenolic compounds in allelopathy has been questioned on several occasions. The main concern is that the levels of phytotoxic compounds in the rice soil are not sufficient to cause phytotoxicity (Olofsdotter et al. 2002). In a previous study, the action of phenolic acids in rice roots exudates was investigated individually or combined finding that, despite rice showed more phytotoxic tolerance to these compounds that arrowhead (*Sagittaria monotevidensis* Cma. & Schltdl), the concentration required for growth inhibition was much higher than the levels found in root exudates (Olofsdotter et al. 2002; Seal et al. 2004). But their participation in allelopathy is still unclear and it has been suggested that phenolic acids might act synergistically with other chemicals to function as allelochemicals. On the contrary, momilactone B has been proposed to be a major allelochemical in rice (Kato-Noguchi et al. 2010).

Previous studies indicated that rice allelopathy is a quantitative genetic trait, which is influenced by environmental conditions. The genetic control of allelopathy has been investigated previously in biparental populations. Quantitative trait loci (QTLs) analysis in a population of PI312777 and Rexmont, using a 215 RFLP markers, revealed seven associated loci distributed in chromosomes 1, 3, 5, 6, 7, 11, and 12 with inhibition of the root length of lettuce plants (Ebana et al. 2001). Additionally, four main QTLs associated to allelopathic activity against barnyardgrass under laboratory conditions were mapped, using 140 DNA markers, in chromosomes 2, 3 and 8 in a population derived from a cross between cultivar IAC 165 (*japonica* variety) and cultivar CO-39 (*indica* variety) (Jensen et al. 2001). In the same way, two QTLS were identified in chromosomes 4 and 7 in a biparental population derived from AC1423, an indica variety with high allelopathic potential and Aus 196, an Aus variety with low allelopathic potential (Jensen et al. 2008). These studies were carried out before the development of the new-generation sequencing techniques. The availability of whole genome sequencing and its low cost, allows the development of high-density SNP-type marker panels that that provide much greater precision in assays than previous ones. In addition, GWAS in a wide diverse population has become an extensive mapping tool to dissect complex agronomic traits. Biparental populations limit the analysis to a relatively low number of genes and, in contrast, GWAS enables to search in a broader gene pool as it is conducted using a wide diversity. Till now, GWAS has not been conducted in rice allelopathy.

Most of the genetic and molecular analyses of the allelopathic potential have been carried out on line PI312777, with strong weed suppressing ability. It is accepted that momilactones (A and B) and phenolics are responsible for the allelopathic potential of this line (Kong et al. 2004, 2006; He et al. 2012). Despite the ability of rice plants to produce diterpenoids that are released into the rhizosphere was recognized decades ago, as well as their function as phytoalexins, the allelopathic activity of these compounds as a natural strategy to prevent weed growth remains relatively unexplored. Particularly there is limited information about allelopathic potential in modern and European varieties and it is not known whether other mechanisms responsible for the allelopathic effect remain to be discovered in other varieties.

In this sense, the search for cultivars adapted to European agroclimatic conditions with the potential to inhibit barnyardgrass, and the elucidation of genes responsible for allelopathy in temperate *japonica* rice remain to be done. In this manuscript, we have investigated allelopathy in a temperate *japonica* rice panel, conducted a GWAS using a high-density panel of SNPs, and assessed the feasibility of initiating a breeding programme to incorporate allelopathy in rice while maintaining grain yield and quality, which will ultimately benefit both farmers and the environment.

## Material and Methods

### Plant Material

A set of 171 accessions showing a wide genetic diversity was generated by selecting a set of 157 accessions from a previously generated rice collection (*Oryza sativa*) (Reig-Valiente et al. 2018) and adding 14 recently released accessions. The set comprises 163 *japonica* and 8 *indica* type accessions.

Barnyard grass seeds were collected from two plants from experimental fields at Tancat de Malta (39° 18’ N 0° 20’ W, Valencia, Spain) and were identified as *Echinochloa crus-galli* and *Echinochloa hispidula.*

### Phenotypic Bioassay

The allelopathic potential of 171 accessions was evaluated using a modified relay seeding technique (Navarez and Olofsdotter 1996). The bioassay was performed in separate batches and replicated twice.

On the first day (Additional file 1: Fig. S1, day 1) 12 rice seeds of each accession were sterilized by immersion in a 2.5% sodium hypochlorite solution for 30 min, followed by rinsing with distilled water thrice. The seeds were placed equidistantly in a 9 cm diameter Petri dish containing two filter papers at the base, a thin layer of perlite at top, watered with 20 ml of MES 1 mM at pH 6.0, and the lid was closed. The seeds were germinated and cultured in a growth chamber with a constant temperature of 28 °C in darkness for three days. On the fourth day (Additional file 1: Fig. S1, day 4) the lids were removed and batches comprising 16 accessions were placed in a plastic box shielded with a transparent plastic cover, to avoid evaporation. Plants were incubated in a growth chamber at 26 °C under a 16 h/8 h (day/night) photoperiod and the plates were watered each day with 3 ml of MES 1 mM pH 6.0. On the same day, sterilized barnyardgrass seeds were germinated in Petri dishes and incubated in a growth chamber at 28 °C under dark conditions for three days. On the seventh day (Additional file 1: Fig. S1, day 7), 9 barnyardgrass seedlings showing root length between 6 and 8 mm were equidistantly placed in small holes in the perlite layer using a forceps in order to keep the roots of the barnyardgrass submerged, avoiding the growth above the surface. As a control, 20 barnyardgrass seedlings were transplanted into an additional Petri dish with perlite, in the absence of rice. All plates were incubated for an additional week.

On the fifteenth day (Additional file 1: Fig. S1, day 15), rice and barnyardgrass (from co-cultured and control plates) root length was measured with a vernier caliper after washing off the perlite. The percentage of inhibition of barnyardgrass root growth was recorded as the difference in root length between barnyardgrass growing in the presence or absence of rice, as shown:

$$\begin{aligned} & {\text{inhibition}}\,{\text{\% }} \\ & \quad = \frac{{{\text{root}}\,{\text{lenght}}\,{\text{barnyardgrass}}\,{\text{control}}\,\left( {{\text{mm}}} \right) - {\text{root}}\,{\text{lenght}}\,{\text{barnyardgrass}}\,{\text{cocultured}}\,\left( {{\text{mm}}} \right)}}{{{\text{root}}\,{\text{lenght}}\,{\text{barnyardgrass}}\,{\text{control}}\,\left( {{\text{mm}}} \right)}} \times 100 \\ \end{aligned}$$

Data filtering, processing, and plotting of phenotype data were conducted by using R statistical software (ver. 4.2.0), employing the packages stats and ggplot2.

### Whole Genome Sequencing and Data Analysis

DNA was extracted from rice fresh leaf tissue using the cetyltrimethylammonium bromide (CTAB) method with slight modifications, as previously described (Murray and Thompson 1980). DNA was quantified using a NanoDrop 1000 Spectrophotometer (Thermo Scientific, Waltham, MA, USA) and Qubit™ dsDNA Quantification Assay Kit using the Qubit 2.0 Fluorometer (Life Technologies, EEUU). Quality was checked by electrophoresis.

Whole Genome Sequencing was performed at Novogene UK Company Limited (Cambridge, United Kingdom). Genomic DNA was randomly sheared into short fragments that were end-repaired, adenylated, and further ligated to Illumina specific indexed paired-end adaptors. The fragments with adapters were PCR amplified, size selected and purified. The final DNA libraries were quantified using Qubit and real-time PCR and a bioanalyzer was used for size distribution detection. The libraries were sequenced using an Illumina NovaSeq 6000 system PE150, according to a 350 bp library type and to standard Illumina operation procedures with a yield of > 8 Gb per lane and median phred quality score of Q36.

BBDuck function, from BBMap software (https://sourceforge.net/projects/bbmap/) was used for adaptor and quality trimming (Forward: 5′-AGATCGGAAGAGCGTCGTGTAGGGAAAGAGTGTAGATCTCGGTGGTCGCCGTATCATT-3′; Reverse: 5′-GATCGGAAGAGCACACGTCTGAACTCCAGTCACGGATGACTATCTCGTATGCCGTCTTCTGCTTG-3′).

Clean reads were aligned to the Nipponbare reference genome (IRGSP-1.0) using BWA-MEM (Li 2013). The Samtools suite (Danecek et al. 2021) was used for downstream processing of alignment files. Specifically, “samtools flagstat” was used to obtain the mapping statistics from each alignment and “samtools view” was deployed to remove unmapped reads and very low-quality alignments (Q < 10).

Multi-sample variant calling was performed with BCFTOOLS “mpileup” and “call” functions to obtain SNPs and INDELs. For each variant, we retained its allelic depth (AD), number of high-quality bases (DP), phred-scaled strand bias P-value (SP), phred-scaled genotype quality (GQ) and posterior genotype probability in Phred scale (GP). BCFTOOLS “view” was used to transform from the bcf format file to a gvcf format and “norm” function for outputting only the first instance when a record is present in multiple lines.

The analysis yielded 351.4 million variants, that were filtered with vcftools using the parameters: --remove-indels, --maf 0.05, --max-missing 0.9, --minQ 30, --minDP 10, --maxDP 30, --max-meanDP 30, --min-meanDP 10, and only 1.0 million SNPs remained.

Additionally, the SNP data matrix was pruned with PLINK software (Purcell et al. 2007) for high levels of pairwise linkage disequilibrium (LD) using “--indep-pairwise 50 5 0.9” (window of 50 SNPs with a shift of 5 SNPs and 0.9 R^2^ cutoff). At this step multiallelic SNPs were also removed. Finally, a 124.019 SNP data array was obtained for the 171 varieties.

### Linkage Disequilibrium (LD) Estimation

The linkage disequilibrium (LD) decay rate was calculated using TASSEL v.5 software (Bradbury et al. 2007) with a sliding window of 50 SNPs, as the chromosomal distance where the Pearson’s correlation coefficient (*r*^2^) between SNP pairs dropped to half its maximum estimated value. Data Plotting was performed with R software v.4.3.0 (R Core Team 2023).

### Principal Component Analysis (PCA)

Principal Component Analysis was performed using “SNPRelate” package (Zheng et al. 2012) from R software. The plot was drawn with the “rgl” package (Murdoch and Adler 2023).

### Population Structure Analysis

The population genetic structure of the collection was assessed using the Bayesian clustering method implemented in STRUCTURE 2.3.4 (Pritchard et al. 2000). For that aim a selection of 18,728 coding SNPs from the 171 varieties was used. To obtain the pool, we intersected the 124,019 filtered SNPs with coding regions (CDS) of the 21,418 IRGSP Nipponbare genes conserved in the whole Rice Gene Index (RGI) collection (*core* genes) (Yu et al. 2023). The RGI collection encompasses 16 platinum standard reference genomes of rice (Zhou et al. 2020). The 18,728 SNP dataset was generated through this intersection process using the bedtools *intersect* function.

The software estimated the optimal number of genetic clusters (K) and calculated each variety’s membership proportion. Analyses were based on the admixture ancestral model for a range of K values from 1 to 7. We performed 10 runs for each K. Each run was implemented with a burn-in period of 10,000 steps followed by 100,000 Monte Carlo Markov Chain iterations. The optimal number of K clusters was estimated with the ad hoc parameter (ΔK) of Evanno (Evanno et al. 2005) in Structure Harvester (Earl and VonHoldt 2012).

Phylogenetic analysis was performed by converting the 124,019 SNP dataset in VCF format into a PHYLIP file using the vcf2phylip.py script (Ortiz 2019). To calculate pairwise genetic distances between rice cultivars a maximum likelihood (ML) method was applied by IQ-TREE v.2.1.2. (Minh et al. 2020) with 1000 bootstrap replicates and the GTR + ASC model, specific for SNP data. Data representation in tree format was displayed with iTOL v.6.8.1. (Letunic and Bork 2021).

### Marker Trait Association Analysis

The relationship between phenotype and the panel of 124,019 SNPs was examined through GWAS using TASSEL v.5 software (Bradbury et al. 2007). A kinship matrix (K) was created from the genotype data utilizing the Centered IBS (Identity-By-State) method, which calculates the probability that alleles randomly drawn from two individuals at the same locus are identical. Loci may consist of one or more nucleotides. In this approach, genotypes are encoded as 2, 1, or 0, corresponding to the count of one of the alleles at that particular locus. As the mean of the percentage of missing data was very low after filtering, 0.08%, then the missing genotype values were replaced with the average genotypic score at that locus before estimating a relationship matrix.

In addition, a distance matrix (Q) of the genotype data was calculated as 1—IBS (Identity-By-State) similarity. From this matrix, a Multidimensional Scaling (MDS) analysis, also known as Principal Co-ordinate Analysis (PcoA), was conducted. The axes produced by MDS were used as covariates to correct the population structure. In addition, batches due to differences in the temporal analysis of the phenotypic analysis were used as covariates.

The MDS results, in conjunction with the genotype (VCF file) and phenotype data (percentage of inhibition), were integrated, and the kinship matrix was incorporated to perform the phenotype-genotype association analysis. For comparison, two statistical models, Mixed Linear Model (MLM) and General Linear Model (GLM), were employed. In the case of MLM, K and Q matrix were used as kinship and population structure controls respectively. Given that MLM offers an effective control of false positives but with potential occasional false negatives, we opted to stablish a threshold value calculated using a non-stringent method, the minimum Bayesian Factor (mBF) (Goodman 2001; Wakefield 2009; Zhang et al. 2019a). The threshold P-value for significant association was set at P ≤ 2.57 × 10^−4^, corresponding to a − log10(P) = 3.59 calculated using the following formula: mBF = −e*P*ln(P).

For GLM, the association analysis was performed using a least squares fixed effects linear model. As GLM may cause potential false positives, the Bonferroni method was used to determine the threshold, given its strength. The threshold for significance was set at P ≤ 8.1 × 10^−6^, corresponding to − log10(P) = 5.09, calculated by the rough Bonferroni correction.

SNPs situated within a region with a distance similar to the LD (considering the calculated LD decay distance of 157 kb) were considered as a single QTL. SNPs displaying the lowest p-value in a QTL were regarded as the leading SNPs. The Manhattan plot was drawn using the R package”qqman”(Turner 2018). Phenotype effect of the leading SNPs was plotted with “ggstatsplot” package (Patil 2021) from the same software.

## Results

### Allelopathic Potential Assay

**Fig. 1:**
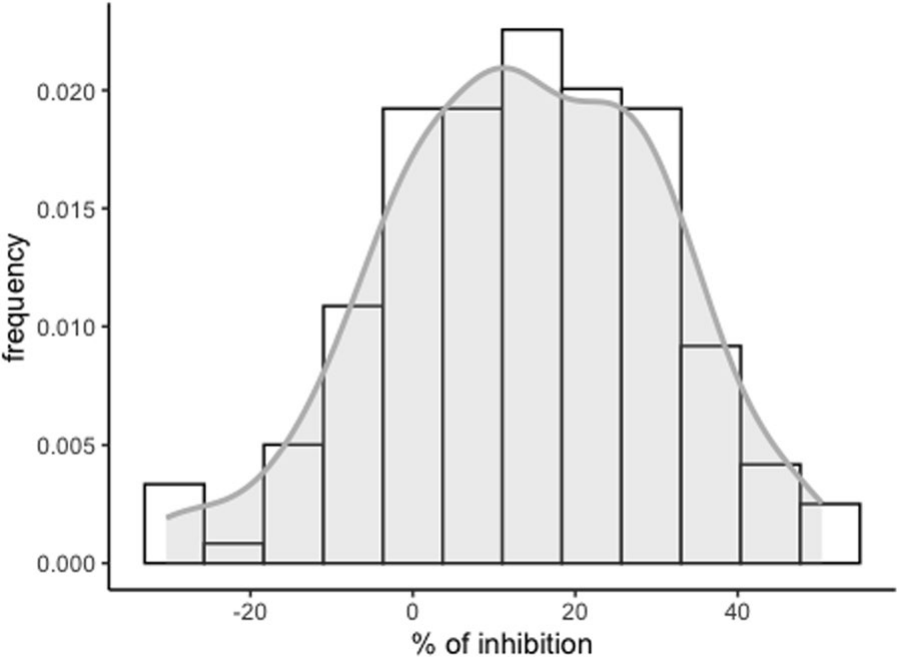
Distribution of barnyardgrass root growth inhibition frequencies. Results obtained from the allelopathic potential bioassay evaluated under coculture conditions with 171 different commercial rice varieties

The ability of inhibition of barnyardgrass root growth was evaluated in plants from a panel of 163 *japonica* varieties adapted to temperate climate (Fig. 1; Additional file 7: Table S1). The collection comprised modern and old varieties as well as some landraces from different geographical origins to cover a wide genetic diversity. The European accessions were the most abundant group, with 105 from four different countries. A set of 8 *indica* cultivars was also included in the collection as a reference of genetic divergence.

Rice seedlings were incubated in the presence of barnyardgrass plants under controlled laboratory conditions for 7 days and then, the length of barnyardgrass roots was measured. We found marked differences in the inhibitory activity among the varieties, with a coefficient of variation of 130.5% indicating the suitability of this collection for the association study. The assay was performed in duplicate and the replicability was evaluated showing a correlation of 0.42 between both replicates (Additional file 2: Fig. S2). Most of the cultivars inhibited the growth of barnyardgrass, among them, 8 varieties suppressed barnyardgrass root growth in 40–50%, representing a 4.7% of the collection. The highest allelopathic potential was exhibited by Ganao, Katy and Senia, inhibiting the barnyardgrass root growth 50.4%, 48.4% and 47.7% respectively compared to plants grown without the presence of rice plants (Additional file 7: Table S1; Additional file 3: Fig. S3). Some varieties showed no inhibition or even a stimulation of barnyardgrass root growth (Additional file 3: Fig. S3). This is the case of Koshihikari, a known variety to exudate momilactone (Kato-Noguchi et al. 2008), that didn’t promote barnyard grass growth stimulation in our conditions. Distribution of root growth inhibition frequencies is shown in Fig. 1.

In addition, the rice root length was measured to find out if the co-cultured could affect the rice root growth. We didn’t find any correlation between the length of barnyardgrass and rice root length, indicating that the inhibition wasn’t caused by space competition (Additional file 2: Fig. S2). Barnyardgrass stem length was also recorded, and no differences in growth were found when cultured in the presence or absence of rice (Additional file 8: Table S2).

### Genome Sequencing and Identification of Polymorphisms

Genome resequencing of the varieties generated a mean of 51 × 10^6^ short reads per cultivar which correspond to 9.53 GB per sample with a mean coverage of 20×. The clean reads were mapped to the Nipponbare reference genome (IRGSP-1.0) and 98% of them were mapped and properly paired. We detected 351 million raw variants in total, which were filtered according to different criteria as call rate > 90% and read coverage between 10× and 30× and quality (see methods). Indels were removed as well as SNPs with a minimum allele frequency (MAF) below 5% were removed. The extent of linkage disequilibrium (LD) in the population was estimated in 157 kbp, when *r*^2^ drops to 0.23 (Additional file 4: Fig. S4). Finally, after SNP pruning by LD, a panel of 124,019 SNPs was obtained, with an average value of 10,334.9 markers per chromosome. Assuming from the newest genome assembly a size of 373 Mpb (Kawahara et al. 2013), the average density was 3.1 kbp/marker, ranging from 2.2 for chromosome 11 to 4.1 for chromosome 4.

### Genetic Structure of the Collection

**Fig. 2:**
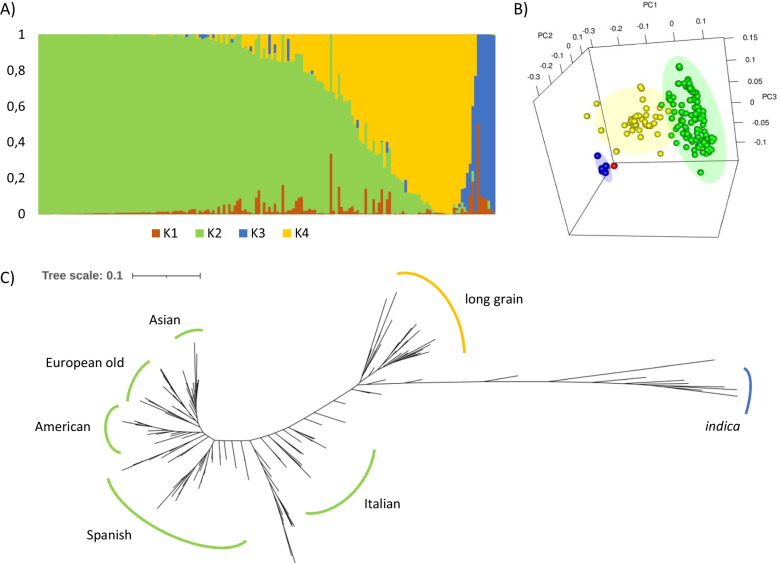
Population structure of the collection based on the genotype data. **A** The estimated membership probability of assigning cultivars to 4 groups. Bar length represents the probability of each variety belonging to different subgroups. **B** 3D Principal Component Analysis (PCA) plots. Three Principal Components are represented as PC1, PC2 and PC3 respectively. **C** Unrooted phylogenetic tree inferred by the maximum likelihood (ML) method. For all nodes, bootstrap percentage was 100. Length branches show the genetic divergence between varieties

Population structure of the collection was estimated using STRUCTURE software. According to this analysis, ∆*K* showed maximum values for K = 4 (∆*K* = 1484.25) indicating that the optimum number of subpopulations was 4 (Additional file 5: Fig. S5). Differentiation of these four subpopulations was corroborated by the Fst values obtained for each group (Additional file 9: Table S3). The collection showed a strong structure with most varieties belonging to two main genetic groups (Fig. 2). While subgroup 1 had limited weight, subgroup 2 included mostly medium grain type cultivars with different geographical origins from Europe, Asia, America and Australia. Subgroup 3 comprises a small group of *indica* accession included in the collection. Finally, long grain type accessions conformed subgroup 4 and displayed also different geographical origins, mainly from Europe and America. To deepen the patterns of population structure, we performed principal components analysis (PCA) that supported two main groups (Fig. 2). The first principal component (PC1) explained the 15.43% of the variance, and the second component (PC2) the 10.97% while the third component (PC3) explained the 4.11% of the genetic variance. These two main PCs separated varieties in accordance with the four subpopulations displayed using STRUCTURE.

The relationship of accessions in the collection and the genetic distances among them were estimated. The distribution of cultivars obtained was roughly in accordance with their origin and grain type in agreement with the population genetic analysis and with previous results (Reig-Valiente et al. 2016). The *indica* accessions, included in subgroup 3 in the population structure analysis, were grouped into a highly supported cluster, and closely, *japonica* long grain type varieties, included in subgroup 4, diverge into an additional cluster (Fig. 2, Additional file 3: Fig. S3). Most of the *japonica* varieties displaying medium grain, included in subgroup 2, were grouped in 5 different clusters with a distribution that clearly matches their origin and, even from some occasions, the release date of the cultivars. Asian and American/Australian varieties were clustered in two different clades, while European accessions were fragmented into several small subgroups that included, mostly, European ancient varieties, Italian and Spanish varieties.

### Association Analysis

**Fig. 3:**
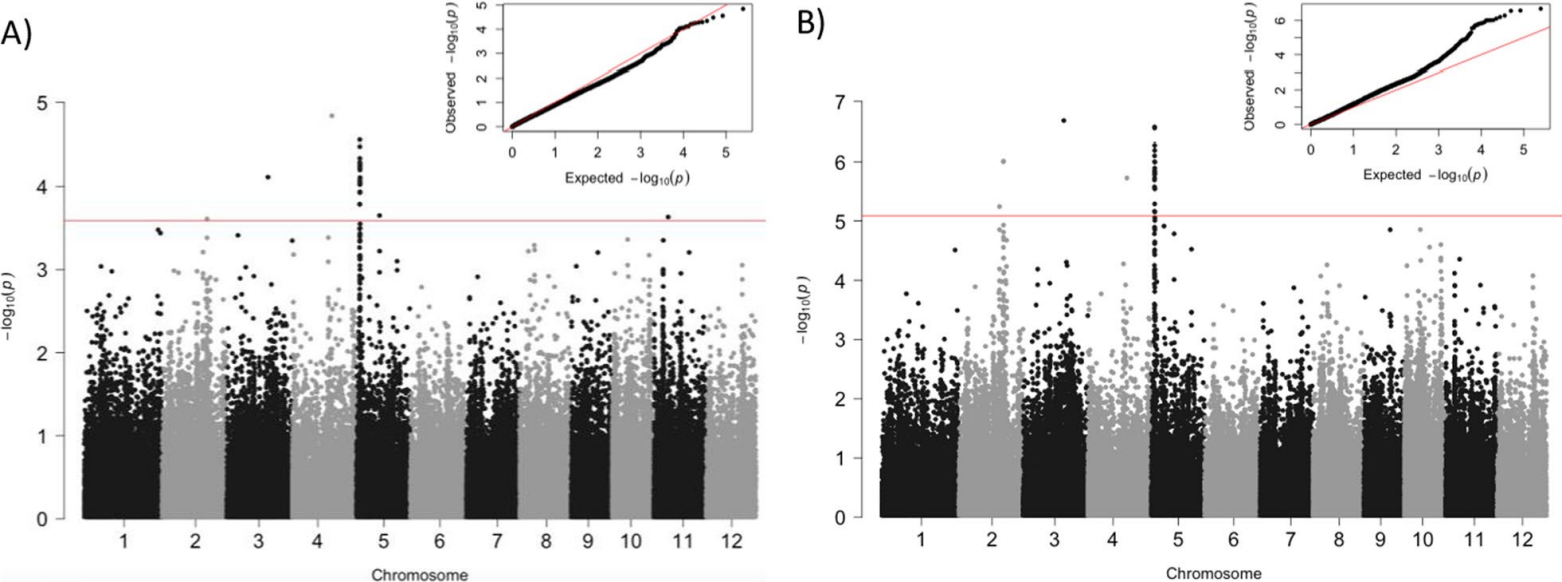
Genome-wide association mapping of the barnyardgrass root growth inhibition. Manhattan and quantile–quantile plots are shown using (**A**) MLM and (**B**) GLM. The red horizontal lines indicate genome-wide significant thresholds

Both Mixed Linear Model (MLM) and General Linear Model (GLM) were used to detect associations between SNP markers and variations in the inhibitory potential of barnyardgrass root growth. It has been suggested that MLM is a stringent method that provides fewer spurious associations than other methods but in contrast increases the number of false negatives (Kaler et al. 2020). Therefore, we used GLM as an additional approach for comparison and verification of the results. P-values and q-values were calculated. In the case of MLM, we set a p-value < 10^−4^ threshold, according to the minimum Bayesian Factor, to consider a SNP as significantly associated with the allelopathic potential. In the case of GLM, as it is a low restrictive method, we set a threshold of p-value < 10^−5^, calculated by the Bonferroni correction test. The q-values were also estimated. The quantile–quantile (QQ) plot and Manhattan plot for the analysed trait obtained using the PCA Qmatrix is shown in Fig. 3. QQ plot indicated that the model was well fitted to the data; the observed p-values were uniformly distributed with some deviation at high values from the expected p-values (Fig. 3).

The statistical analysis revealed several SNPs significantly associated with barnyardgrass root growth inhibition (Table 1; Additional file 10: Table S4), which corresponded to 4 QTLs, considering an association locus as a chromosomal region in which the distance between the adjacent pairs of associated SNPs is given by the estimated LD. qAll-2a and qAll-2b, only detected using GLM, were located in chromosome 2 at positions 22,671,912 and 24,842,411, respectively. qAll-2b consisted in three SNPs located in a region of 64.1 Kb. qAll-3 was located in chromosome 3 at position 22,755,757. qAll-5, in chromosome 5, comprises a region of 141.1 Kb or 194.6 Kb depending on the applied methodology displaying a peak value at position 1,843,023, given by the lowest p-value (Table 1). An additional single SNP above the threshold using both methods was found on chromosome 4 at position 21,850,604, which was excluded because there were no significant SNPs located nearby and to the undetermined allele content in the accessions showing differential root growth inhibition (Additional file 6: Fig. S6).

**Table 1:** List of lead-SNPs significantly associated with the barnyardgrass root growth inhibition (MLM, p-value < 10^−4^; GLM, p-value < 10^−5^)

QTL	Chr	Lead SNP position	MLM			GLM		
			p-value	Significant SNPs	Genomic interval	p-value	Significant SNPs	Genomic interval
qAll-2a	2	22.671.912				5.69E−06	1	
qAll-2b	2	24.870.799				9.71E−07	3	64.115
qAll-3	3	22.755.757	7.71E−05	1		2.10E−07	1	
qAll-5	5	1.843.023	2.80E−05	16	141.090	269E−07	20	194.608

**Fig. 4:**
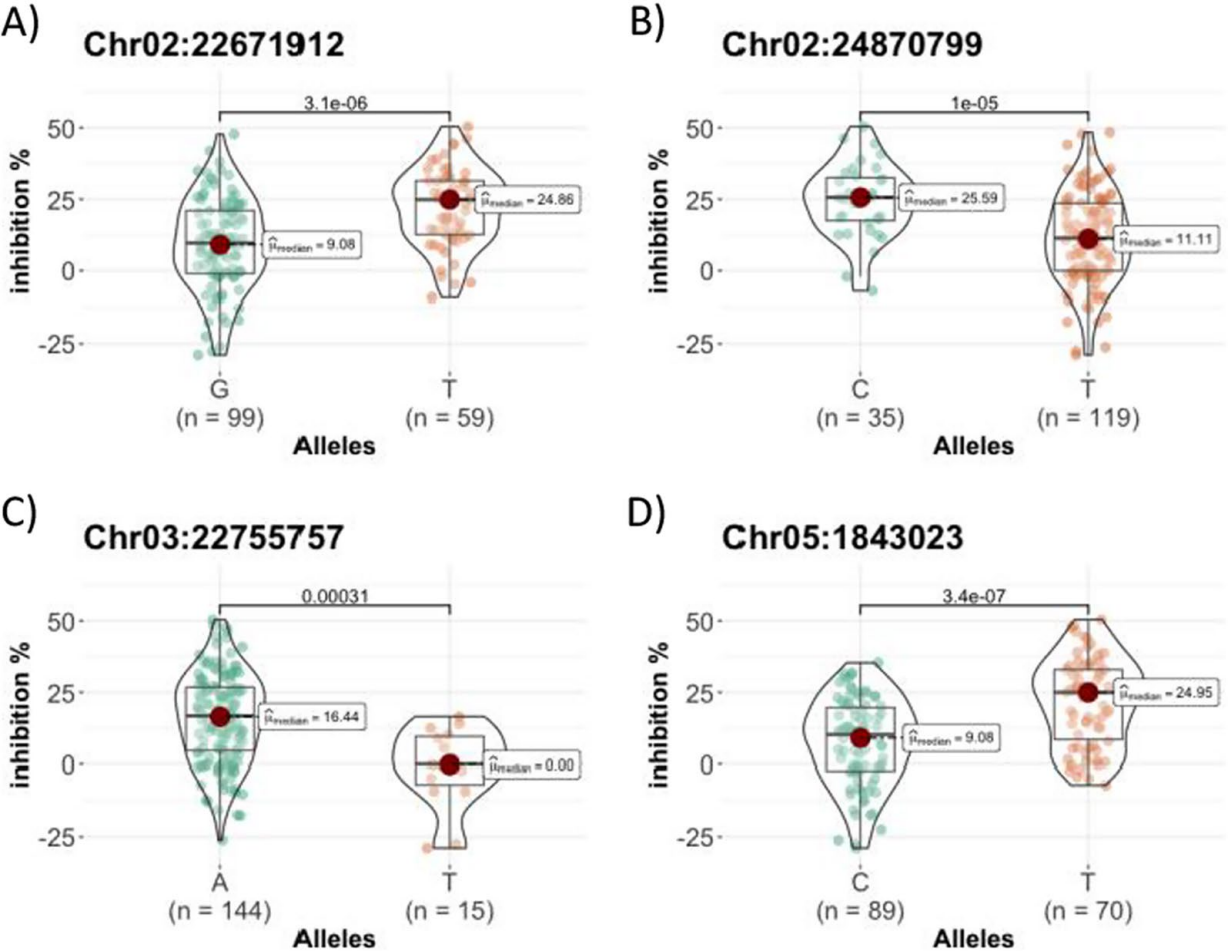
Violin plots. Comparison of the allele content at the lead SNP **A** qAll-2a, **B** qAll-2b, **C** qAll-3 and **D** qAll-5 in the population

We analyzed the allele content in the lead SNP and found that they displayed promising allele distribution among the cultivars as significative differences in the growth inhibition ability according to allele present in the accessions were found in all identified QTLs (Fig. 4). Accessions carrying different alleles of SNP qAll-2 showed a different barnyardgrass root growth inhibition value, those carrying T displayed 24.86% in contrast to those carrying G which displayed 9.08%. Similarly, qAll-2b, accessions carrying the minor allele C showed a root growth inhibition of 25.59%, meanwhile, those accessions carrying the major allele T showed 11.11% inhibition. While accessions carrying allele T in qAll-3 lead SNP didn’t show barnyardgrass root inhibition, plants with the most common allele A showed a root growth inhibition of 16.4%. The mean values for barnyardgrass root growth inhibition were lower in the accession containing C, 9.08% of inhibition, than those carrying T, 24.95% of inhibition, at the lead SNP in qAll-5. The analysis of Ganao allele content in the lead marker of the four QTLs revealed that they carried the favourable allelopathy allele.

### Mining Candidate Genes for Allelopathy

We looked for annotated genes that could be involved in allelopathic activity within the four QTL regions in an extended the interval of 150 Kb, using the annotated reference genome Nipponbare IRGSP-1.0 (RAP-DB) (Table 2).

**Table 2:** List of known genes potentially related to allelopathy within 0.15 Mb distance interval from each QTL lead marker

Locus ID	Description	Gene symbol	QTL	Reference
*Os05g0132700*	R2R3-MYB TRANSCRIPTION FACTOR 52	*2R_MYB52*	4	
*Os02g0623400*	G1 LIKE PROTEIN 3	*G1L3*	2	Sultana et al (2023), Fang et al. (2009)
*Os02g0624300*	R2R3-type MYB transcription factor	*MYB30*	2	
*Os02g0626100*	Phenylalanine ammonia-lyase 1	*OsPAL1*	2	
*Os02g0626400*	Phenylalanine ammonia-lyase	*OsPAL*	2	
*Os02g0626600*	phenylalanine ammonia-lyase 3	*OsPAL3*	2	Sultana et al. (2023)
*Os02g0627100*	Phenylalanine ammonia-lyase 4	*OsPAL4*	2	
*Os02g0589000*	Lecithin:cholesterol acyltransferase family protein		1	Sultana et al. (2023)
*Os02g0589700*	Lecithin:cholesterol/phospholipid:diacylglycerol acyltransferase domain containing protein		1	
*Os02g0590000*	Lecithin:cholesterol/phospholipid:diacylglycerol acyltransferase domain containing protein		1	
*Os02g0590200*	Lecithin:cholesterol/phospholipid:diacylglycerol acyltransferase domain containing protein		1	
*Os02g0590400*	Lecithin:cholesterol acyltransferase family protein		1	

*Os02g0589000*, coding for a lecithin:cholesterol acyltransferase, was localised in qAll-1. Lecithin:cholesterol acyltransferases, also named as phospholipid:diacylglycerol acyltransferases, are involved in the synthesis of triacylglycerol (Dahlqvist et al. 2000). The up-regulation of this gene has been observed in previous allelopathic assays (Sultana et al. 2023). Four additional genes coding for proteins of the lecithin:cholesterol acyltransferases family were localised in the vicinity in qAll-2. Another gene involved in lipid metabolism, *Os05g0133401*, coding for an esterase, is localised in qAll-5 (Additional file 11: Table S5).

PAL is a key enzyme in the phenylpropanoid synthesis pathway and, it has been related to allelopathic activity on barnyard grass in several occasions (Fang et al. 2013). Four genes coding for PAL could be found in qAll-2b. One of these genes *Os02g0626600*, coding for PAL3, was previously found to be up-regulated in response to the presence of barnyard grass (Song et al. 2008; Sultana et al. 2023).

Next to qAll-2b SNP peak, *G1L3* is also localised. The up-regulation of *G1 LIKE PROTEIN 3* (*G1L3*) has been reported previously in transcriptomic analysis of rice in the interaction with barnyardgrass (Fang et al. 2009; Sultana et al. 2023).

Two genes coding for R2R3-type MYB transcription factors, MYB30 and MYB52, were found located in qAll-2b and qAll-5. R2R3-MYB factors play a positive role in the regulation of genes involved in secondary metabolism (Fang et al. 2020). It has been suggested that *OsMYB30* is a regulator of root cell elongation under ROS signals (Mabuchi et al. 2018) and root hair elongation under stress in Arabidopsis (Xiao et al. 2021). More interestingly, *OsMYB30* has been implicated in the biosynthesis of the phenolic compound stilbene in grapevine (Mu et al. 2023).

qAll-5 covers a genomic area that comprises 21 annotated genes (Additional file 11: Table S5). *Os05g0131500*, coding for a ferroportin was localised next to the leader marker position, but the role of this protein in allelopathy has not been reported previously (Additional file 11: Table S5).

No genes identified previously as associated with allelopathy have been detected in the region near qAll-3 (Additional file 11: Table S5).

## Discussion

The management of damaging weeds in rice cultivation poses a significant challenge across various regions. Efforts to control these weeds while minimizing herbicide use are highly desirable, as this approach can help prevent the development of herbicide resistance and contribute to cost reduction in crop production. Some rice varieties can exhibit allelopathy and it has emerged as a potential solution for weed management in rice fields. When barnyardgrass seedlings were co-cultivated with specific rice varieties, they exhibited a reduction in root growth, highlighting the potential of allelopathy as a promising avenue for weed control in rice cultivation.

In this study, we examined the allelopathic potential of 171 *japonica* rice varieties by assessing their ability to inhibit the growth of barnyardgrass roots when co-cultured. Allelopathy can be induced by several factors as stress or nutrient deficiency (Song et al. 2008). It has been demonstrated that plant defense signalling hormones, such as jasmonic acid or salicylic acid, can induce allelopathy (Fang et al. 2009). To avoid inductor factors affecting the allelopathic activity, the bioassays were performed under controlled laboratory conditions. We performed analysis at early developmental stages as our goal was to find rice varieties with allelopathic potential, and the genes responsible for this potential, to improve the ability of rice seedlings to resist paddy weeds as early as possible in the field to provide a competitive advantage for rice by inhibiting the growth of competing weeds. Our findings revealed a substantial variation in the inhibition of barnyardgrass root growth when co-cultured with the different varieties. Within this collection, we identified novel allelopathic potential in varieties, as Ganao.

We employed GWAS to uncover genetic variants linked to the interaction between rice and barnyardgrass. The varieties we investigated were part of a previously established *japonica* collection, which encompasses those typically grown in temperate climates (Reig-Valiente et al. 2018). This collection has previously been utilized successfully, using a panel of 1713 SNPs, to identify genetic polymorphisms associated with various agronomic traits and serves as a valuable resource for pinpointing genetic factors responsible for the variations in agronomic traits within these regions (Reig-Valiente et al. 2018). In this case, to facilitate a more precise phenotypic-genotypic association analysis, we conducted whole-genome resequencing of the selected rice varieties, enabling the creation of a high-density SNP panel. As a result, we used a high-density SNP panel and identified four QTLs as candidate to play a role in the interaction between barnyardgrass and rice in *japonica* temperate rice.

Among these QTLs, qAll-2b stands out as it encompasses a cluster of four PAL coding genes associated with phenylpropanoid synthesis, which are compounds known to be involved in allelopathy. The first steps in the synthesis of phenylpropanoid-derived compounds are catalyzed by PAL, cinnamate 4-hydroxylase (C4H), and p-coumaroyl coenzyme A ligase (4CL). In this context PAL plays a pivotal role in the phenylpropanoid pathway, responsible for synthesizing various secondary metabolites. PAL facilitates the deamination of phenylalanine, an essential amino acid, to produce trans-cinnamic acid, a crucial precursor for a diverse array of phenolic compounds, including flavonoids, lignin, and other phenylpropanoid derivatives. These compounds are integral to the allelopathic properties of rice. Earlier studies have also highlighted the positive regulatory role of PAL in rice's allelopathic potential (Fang et al. 2013; Zhang et al. 2019b). PAL exhibits up-regulation in hydroponic systems in response to barnyard grass roots (Zhang et al. 2019b; Sultana et al. 2023). Additionally, when the expression of *OsPAL* is silenced, it results in reduced allelopathic activity from donor rice to barnyard grass (Fang et al. 2020). Notably, *PAL* is part of a multi-gene family in plants, with four members located on chromosome 2, and these were identified in qAll-2b.

In our association analysis, we detected two R2R3-MYB transcription factors within qAll-2b and 5. Prior studies have demonstrated the role of MYB transcription factors in regulating genes involved in the plant phenylpropanoid metabolic pathway. Specifically, R2R3-MYB factors are known to govern genes related to secondary metabolism (Fang et al. 2020). For instance, MYB57, an R2R3-MYB transcription factor, has been shown to transcriptionally regulate *MAPK11*, which interacts with PAL2;3 and modulates rice allelopathy (Fang et al. 2020). MYB30, on the other hand, is implicated in root hair development and functions within a MYB30-EIN3 antagonistic module in Arabidopsis (Xiao et al. 2021).

qAll-5 represents a particularly promising locus, housing a genomic region containing 21 genes. While some of these genes may have a role in allelopathy as *2R_MYB52*, others, such as ferroportin found next to the lead marker position, have not been previously associated with this context. Additional investigation is required to elucidate their involvement in allelopathy.

Contents of allelochemicals in rice have been known to be varietal dependent (Kato-Noguchi et al. 2010). Several secondary metabolites have been found in root exudates that include phenolic acids, terpenes, fatty acids and indoles that have been considered as potential allelochemicals (Macías et al. 2007). Among them, momilactone B is present in roots exudates from different varieties and it has been proposed that the allelopathic activity of rice may depend primarily on the secretion level of momilactone B (Kato-Noguchi et al. 2010). The highly allelopathic potential line PI312777 also secretes momilactone B in the root exudates when cocultured with barnyadgrass. The GWAS analysis of the *japonica* temperate collection identified candidate genes for allelopathy that were related to phenolic acid and lipid metabolism, but not genes directly involved in the synthesis of momilactones were found. Our findings point to the participation of phenolic acids in allelopathy. Whether they are sufficient or they need to be accompanied by other chemicals to produce allelopathy, needs to be investigated.

Previous QTL analysis was conducted using RFLP markers in a bi-parental population resulting from the cross between PI31277 and Rexmont (Ebana et al. 2001). This analysis identified seven QTLs, with one of them located on chromosome 5, approximately 0.65 Mb away from qAll-5. Our use of a diverse panel of rice varieties, combined with genome sequencing techniques, significantly expands the potential to pinpoint QTLs associated with allelopathy with a higher degree of precision. Given the lower precision of the previously utilized technique compared to the use of a high-density panel of SNPs for GWAS analysis, it is highly likely that both QTLs are the same. The fact that this QTL has been identified in two studies, one of which employed a wide genetic diversity, strengthens the case for the significant role of this genomic region in allelopathy. Moreover, a detailed examination of the allelic content in the leader marker of qAll-5 revealed that 70 varieties carrying one allele inhibited *Echinochloa* root growth by 24.9%, in contrast to 9.1% for the 89 varieties carrying the other allele. qAll-5 emerges as a promising candidate for genetic breeding focused on enhancing allelopathy in rice.

## Conclusions

We have assessed the allelopathic potential of a collection of *japonica* rice adapted to temperate regions and identified varieties that can inhibit the root growth of barnyardgrass when co-cultured. The association analysis using a high-density panel of markers mapped 4 QTLs that may help to understand allelopathy mechanism of action. These QTLs indicate that the phenylpropanoid synthesis pathway is taken part in the allelopathic potential of varieties within this collection. Among the identified QTLs, qAll-2b and qAll-5 are robust and constitute candidates to initiate a breeding program. The discovery of rice genotypes possessing allelopathic potential against weeds, along with the corresponding identification of associated QTLs, opens the opportunity to initiate a breeding program to incorporate allelopathy in rice while maintaining grain yield and quality that, ultimately, will benefit farmers as well as the environment as a powerful tool to combat parasitic weeds providing new weed management strategies in agriculture.

### Supplementary Information

**Additional file 1. Fig. S1**: Rice sterilized seeds by immersion in a 2.5% sodium hypochlorite solution for 30 min on the first day. The seeds were placed equidistantly in Petri dishes containing a layer of perlite and 20 ml MES 1 mM, pH 6.0. On the fourth day, batches comprising 16 accessions were placed in a plastic box shielded with a transparent plastic cover and incubated in a growth chamber. On the same day, sterilized barnyardgrass seeds were germinated in Petri dishes and incubated in a growth chamber. On the seventh day, barnyardgrass seedlings showing 6–8 mm root were equidistantly placed in small holes in the perlite layer. As a control, barnyardgrass seedlings were incubated in the absence of rice. All plates were incubated for an additional week. On the fifteenth day, root length of rice and barnyardgrass plants (from co-cultured and control plates) was measured.

**Additional file 2. Fig. S2**: (A) Correlation between the root inhibition observed between replicates. (B) Correlation between rice and barnyardgrass root length of plants from a subset of the collection.

**Additional file 3. Fig. S3**: Neighbour-Joining tree of 171 rice accessions. The growth inhibition ability is indicated in grey bars, the group structure membership is indicated in circles for each variety where the proportion of membership to each group is indicated with colours. The country of origin of each variety is shown.

**Additional file 4. Fig. S4**: Estimated LD decay in the collection, expressed as decay of r^2^.

**Additional file 5. Fig. S5**: Estimated Delta K (∆*K*) based on the STRUCTURE analysis.

**Additional file 6. Fig. S6**: Violin plot. Comparison of the allele content at position 21850604 on chromosome 4.

**Additional file 7. Table S1**: List of cultivars included in the analysis with their country of origin, inhibition percentage of barnyardgrass root inhibition. Membership to K groups defined by STRUCTURE is indicated.

**Additional file 8. Table S2**: Stem length of barnyardgrass plants co-cultured with different rice varieties. Stem lengths of plants grown in the presence of rice were compared with plants grown without rice and the p-value was calculated. Statistical analysis is shown.

**Additional file 9. Table S3**: Mean Fst values obtained for each structure group.

**Additional file 10. Table S4**: List of SNPs significantly associated with the barnyardgrass root growth inhibition.

**Additional file 11. Table S5**: List of annotated genes within 150 kb distance interval from the SNP marker peak.

## Data Availability

The datasets supporting the conclusions of this article are available in the European Nucleotide Archive with the primary accession code PRJEB71817.

## Abbreviations

GLM: General linear model
GWAS: Genome Wide Association Study
MLM: Mixed linear model
PAL: Phenylalanine Ammonia Lyase
PCA: Principal components analysis
QTL: Quantitative trait loci
