# Supplementary figures and images for: Deciphering the Genetic Basis of Allelopathy in japonica Rice Cultivated in Temperate Regions Using a Genome-Wide Association Study
Source: Rice (N Y). 2024 Mar 26;17:22. doi: 10.1186/s12284-024-00701-3 (PMC10965883; doi:10.1186/s12284-024-00701-3)

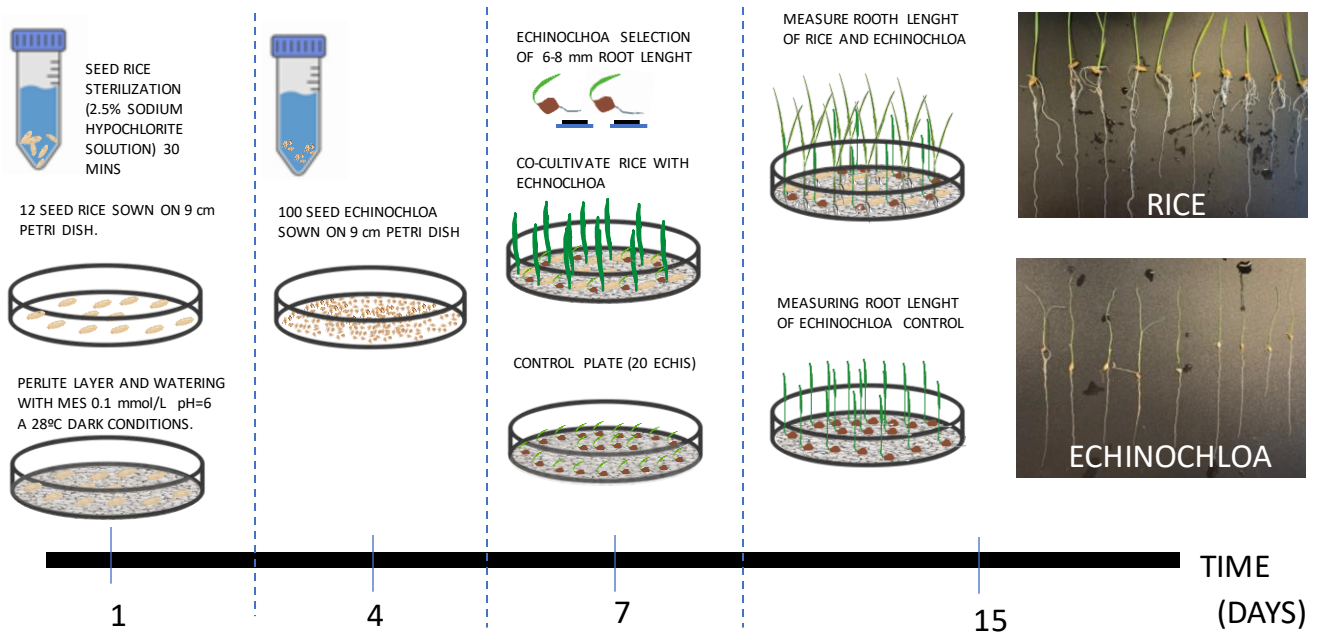

**Supplementary Figure S1.-**

Supplement: Supplementary file 1 — Additional file 1. Fig. S1: Rice sterilized seeds by immersion in a 2.5% sodium hypochlorite solution for 30 min on the first day. The seeds were placed equidistantly in Petri dishes containing a layer of perlite and 20 ml MES 1 mM, pH 6.0. On the fourth day, batches comprising 16 accessions were placed in a plastic box shielded with a transparent plastic cover and incubated in a growth chamber. On the same day, sterilized barnyardgrass seeds were germinated in Petri dishes and incubated in a growth chamber. On the seventh day, barnyardgrass seedlings showing 6–8 mm root were equidistantly placed in small holes in the perlite layer. As a control, barnyardgrass seedlings were incubated in the absence of rice. All plates were incubated for an additional week. On the fifteenth day, root length of rice and barnyardgrass plants (from co-cultured and control plates) was measured. [file 12284_2024_701_MOESM1_ESM.pdf]

A)

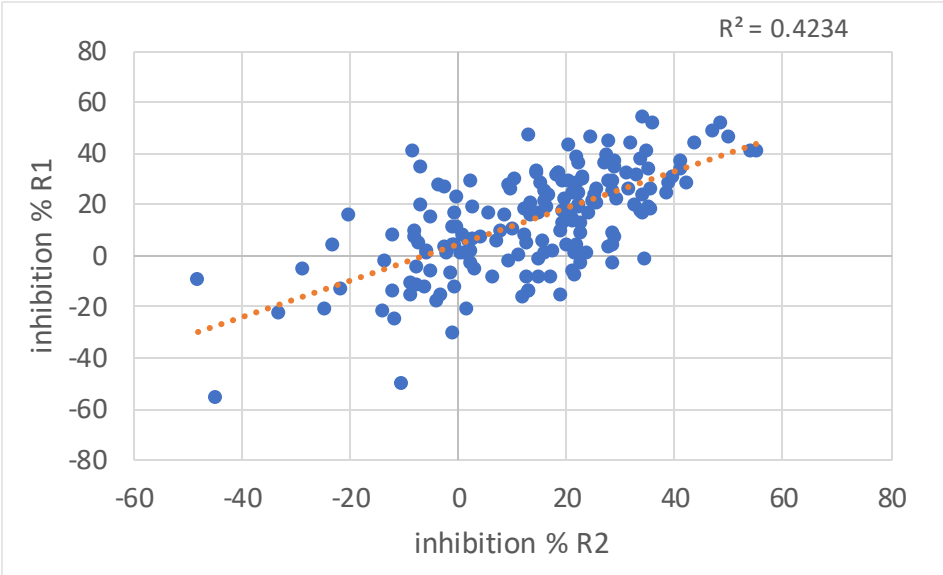

B)

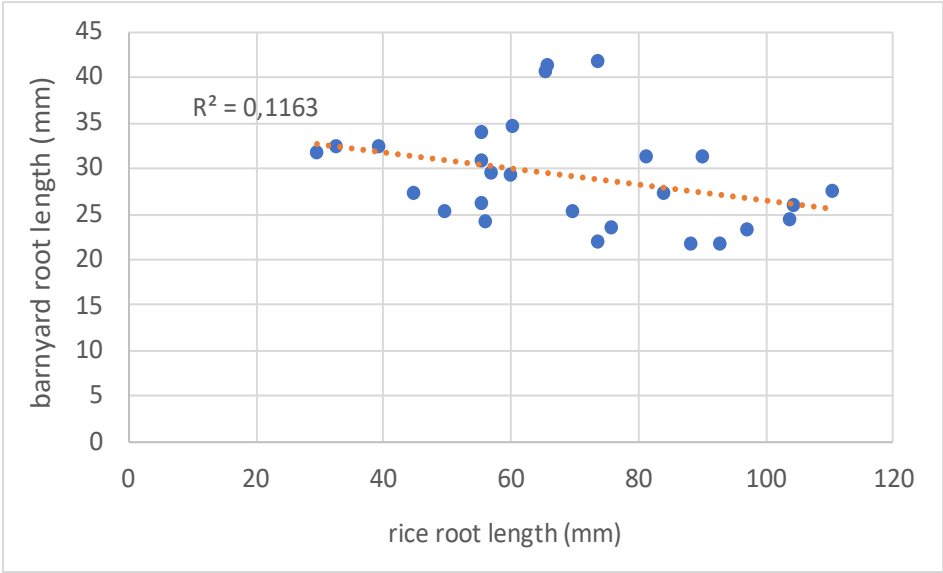

Supplementary Figure S2.

Supplement: Supplementary file 2 — Additional file 2. Fig. S2: (A) Correlation between the root inhibition observed between replicates. (B) Correlation between rice and barnyardgrass root length of plants from a subset of the collection. [file 12284_2024_701_MOESM2_ESM.pdf]

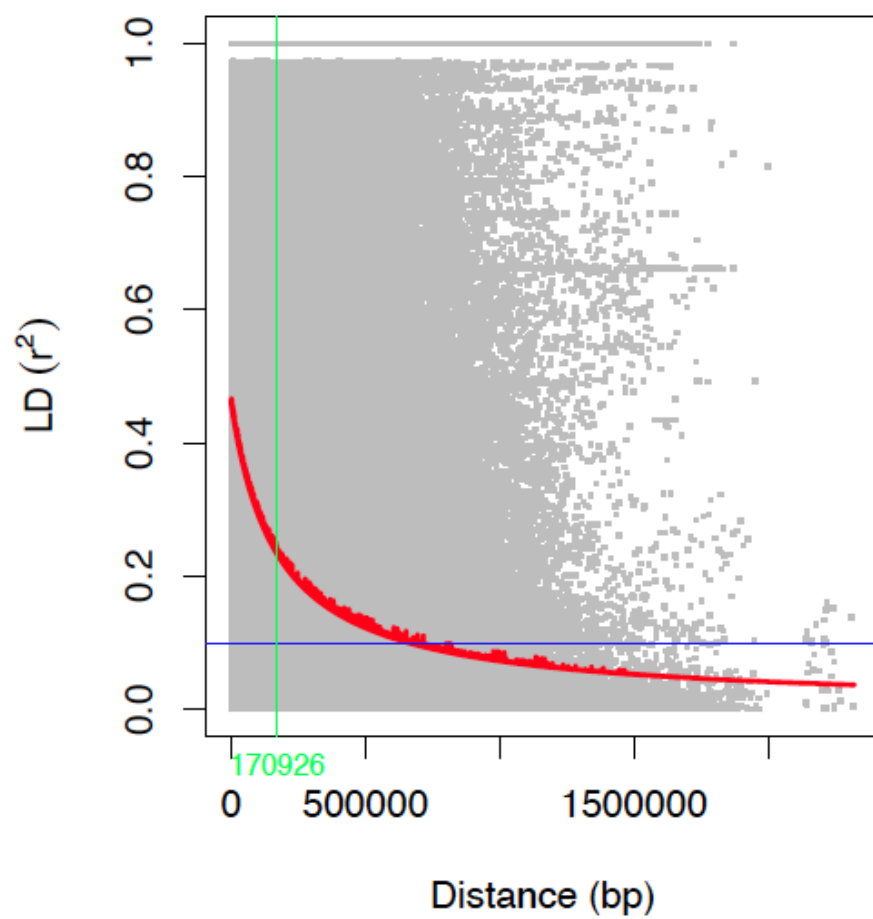

**Supplementary Figure S4.** Estimated LD decay in the collection, expressed as decay of  $r^2$ .

Supplement: Supplementary file 4 — Additional file 4. Fig. S4: Estimated LD decay in the collection, expressed as decay of r2. [file 12284_2024_701_MOESM4_ESM.pdf]

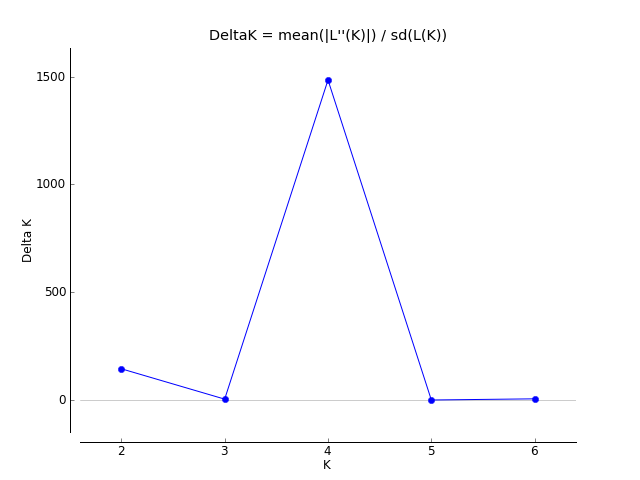

Supplement: Supplementary file 5 — Additional file 5. Fig. S5: Estimated Delta K (∆K) based on the STRUCTURE analysis. [file 12284_2024_701_MOESM5_ESM.png]

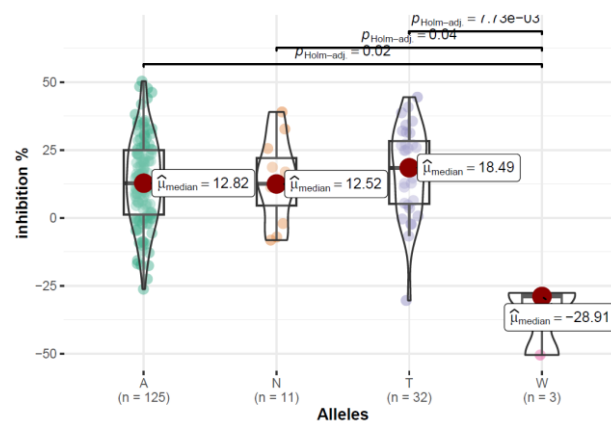

Supplementary Figure S6.

Supplement: Supplementary file 6 — Additional file 6. Fig. S6: Violin plot. Comparison of the allele content at position 21850604 on chromosome 4. [file 12284_2024_701_MOESM6_ESM.pdf]
