# Supplementary material for: Deciphering the Genetic Basis of Allelopathy in japonica Rice Cultivated in Temperate Regions Using a Genome-Wide Association Study
Source: Rice (N Y). 2024 Mar 26;17:22. doi: 10.1186/s12284-024-00701-3 (PMC10965883; doi:10.1186/s12284-024-00701-3)
Supplement: Supplementary file 3 — Additional file 3. Fig. S3: Neighbour-Joining tree of 171 rice accessions. The growth inhibition ability is indicated in grey bars, the group structure membership is indicated in circles for each variety where the proportion of membership to each group is indicated with colours. The country of origin of each variety is shown. [file 12284_2024_701_MOESM3_ESM.pdf]

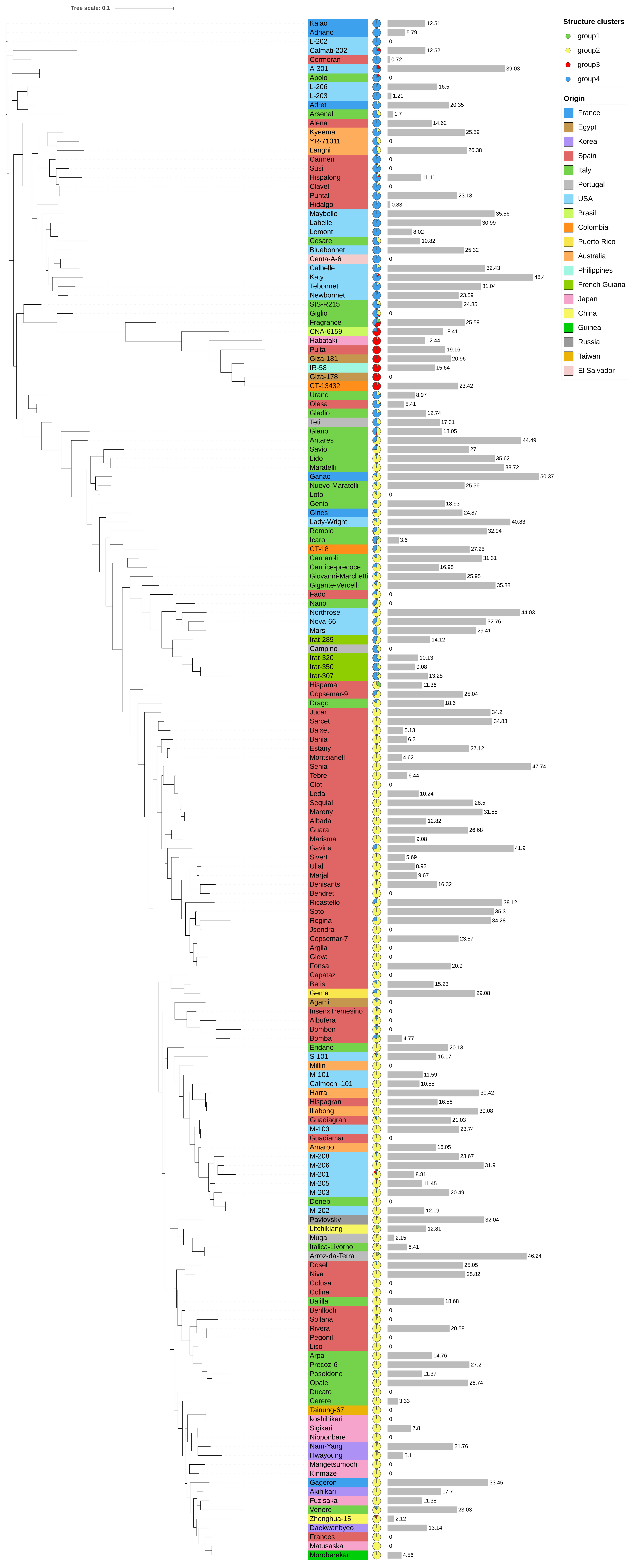
Tree scale: 0.1
Structure clusters
group1
group2
group3
group4
Origin
France
Egypt
Korea
Spain
Italy
Portugal
USA
Brasil
Colombia
Puerto Rico
Australia
Philippines
French Guiana
Japan
China
Guinea
Russia
Taiwan
El Salvador
Kalao 12.51
Adriano 5.79
L-202 0
Calmati-202 12.52
Cormoran 0.72
A-301 39.03
Apolo 0
L-206 16.5
L-203 1.21
Adret 20.35
Arsenal 1.7
Alena 14.62
Kyeema 25.59
YR-71011 0
Langhi 26.38
Carmen 0
Susi 0
Hispalong 11.11
Clavel 0
Puntal 23.13
Hidalgo 0.83
Maybelle 35.56
Labelle 30.99
Lemont 8.02
Cesare 10.82
Bluebonnet 25.32
Centa-A-6 0
Calbelle 32.43
Katy 48.4
Tebonnet 31.04
Newbonnet 23.59
SIS-R215 24.85
Giglio 0
Fragrance 25.59
CNA-6159 18.41
Habataki 12.44
Puita 19.16
Giza-181 20.96
IR-58 15.64
Giza-178 0
CT-13432 23.42
Urano 8.97
Olesa 5.41
Gladio 12.74
Teti 17.31
Giano 18.05
Antares 44.49
Savio 27
Lido 35.62
Maratelli 38.72
Ganao 50.37
Nuevo-Maratelli 25.56
Loto 0
Genio 18.93
Gines 24.87
Lady-Wright 40.83
Romolo 32.94
Icaro 3.6
CT-18 27.25
Carnaroli 31.31
Carnice-precoce 16.95
Giovanni-Marchetti 25.95
Gigante-Vercelli 35.88
Fado 0
Nano 0
Northrose 44.03
Nova-66 32.76
Mars 29.41
Irat-289 14.12
Campino 0
Irat-320 10.13
Irat-350 9.08
Irat-307 13.28
Hispamar 11.36
Copsemar-9 25.04
Drago 18.6
Jucar 34.2
Sarcet 34.83
Baixet 5.13
Bahia 6.3
Estany 27.12
Montsianell 4.62
Senia 47.74
Tebre 6.44
Clot 0
Leda 10.24
Sequial 28.5
Mareny 31.55
Albada 12.82
Guara 26.68
Marisma 9.08
Gavina 41.9
Sivert 5.69
Ullal 8.92
Marjal 9.67
Benisants 16.32
Bendret 0
Ricastello 38.12
Soto 35.3
Regina 34.28
Jsendra 0
Copsemar-7 23.57
Argila 0
Gleva 0
Fonsa 20.9
Capataz 0
Betis 15.23
Gema 29.08
Agami 0
InsenxTremesino 0
Albufera 0
Bombon 0
Bomba 4.77
Eridano 20.13
S-101 16.17
Millin 0
M-101 11.59
Calmochi-101 10.55
Harra 30.42
Hispagran 16.56
Illabong 30.08
Guadiagran 21.03
M-103 23.74
Guadiamar 0
Amaroo 16.05
M-208 23.67
M-206 31.9
M-201 8.81
M-205 11.45
M-203 20.49
Deneb 0
M-202 12.19
Pavlovsky 32.04
Litchikiang 12.81
Muga 2.15
Italica-Livorno 6.41
Arroz-da-Terra 46.24
Dosel 25.05
Niva 25.82
Colusa 0
Colina 0
Balilla 18.68
Benlloch 0
Sollana 0
Rivera 20.58
Pegonil 0
Liso 0
Arpa 14.76
Precoz-6 27.2
Poseidone 11.37
Opale 26.74
Ducato 0
Cerere 3.33
Tainung-67 0
koshihikari 0
Sigikari 7.8
Nipponbare 0
Nam-Yang 21.76
Hwayoung 5.1
Mangetsumochi 0
Kinmaze 0
Gageron 33.45
Akihikari 17.7
Fuzisaka 11.38
Venere 23.03
Zhonghua-15 2.12
Daekwanbyeo 13.14
Frances 0
Matusaska 0
Moroberekan 4.56
